# Applying a Pedestrian Level of Service in the Context of Social Distancing: The Case of the City of Madrid

**DOI:** 10.3390/ijerph182111037

**Published:** 2021-10-20

**Authors:** Ruben Talavera-Garcia, Rocío Pérez-Campaña

**Affiliations:** tGIS Research Group, Geography Department, Complutense University of Madrid, 28040 Madrid, Spain; r.perez.campana@ucm.es

**Keywords:** walking, level-of-service, COVID-19, pedestrian mobility, smart cities, tactical urbanism

## Abstract

During the COVID-19 pandemic, there has been a lot of discussion about keeping interpersonal distance to prevent the virus from spreading. To keep this interpersonal distance, authorities at different levels have taken measures to reduce people’s interactions, such as reducing capacities, curfews, pop-up cycle lanes, temporary pedestrianisation, and lockdowns. Many of these temporary measures have been perceived from a static view. Nevertheless, in a scenario of “new normality” or in the face of a possible new pandemic, the amount of data (big data) generated by different sources, such as sensors, in large cities has extraordinary potential to be used together with tactical urbanism for quick adaptation. The aim of this study was to gain insight into the aforementioned issues by analysing spatio-temporal patterns of pedestrian mobility and developing a variation of the pedestrian level of service measure; the pandemic pedestrian level of service (P-PLOS). This measure provides a dynamic view of pavement capacities according to the interpersonal distance recommendations during the pandemic. P-PLOS was tested in the city of Madrid based on the pedestrian counter data that was provided by the local government through its open data website. We found that the application of P-PLOS, together with street design, allows for knowing where and when it is necessary to take tactical urbanism measures in order to maintain or improve the level of service, as well as where it is necessary to take measures to reduce pedestrian flow.

## 1. Introduction

With the arrival of COVID-19, the scientific community and public bodies began to ask people to practice social distancing as one of the main measures to prevent contagion [1]. These measures to reduce virus transmission have led to a reduction in mobility, to a greater or lesser extent, at the international level [2,3,4,5], as well as a change in daily mobility patterns [6]. However, the impact on mobility has not been the same for different modes of transport. In the case of bicycles, it has led to an increase in the use of bicycles as a mode of transport due to the distance it allows and the implementation of numerous temporary bicycle lanes by the public bodies [7]. Nevertheless, the use of private vehicles has increased, to the detriment of public transport [8].

With the recommendations for social distancing and restrictions on mobility, it gradually became clear that it was difficult to maintain the distance in some situations and environments, in particular, when pavements are narrower than the minimum recommended distance between two or more people. In fact, the early months of the pandemic saw the appearance of a slew of websites (e.g., https://www.sidewalkwidths.nyc (accessed on 5 April 2021)), blog entries, and applications dedicated to the analysis of the width of pavements in relation to the recommended social distance.

However, the lack of tools for monitoring and controlling pedestrian mobility has become evident. In this respect, it should be recalled that the scholarly research on the safety of human crowds has mainly been developed within the last few years [9]. This is due, among other factors, to technological advances related to the detection of moving objects in general and moving people in particular [10]. Within the context of COVID-19, this lack of tools is, in turn, due to the lack of data acquisition devices and uncertainty about the spread of the virus. Concerning data acquisition, the existing literature shows that pedestrian dynamics are mainly analysed through empirical methods and visual crowd analysis, including simulations [9]. The use of real data, e.g., from pedestrian counters, appears to be much lower, as this technology is quite recent and not many cities utilise them. Regarding the spread of the virus, this depends on different variables, including droplet size [11], air temperature, air humidity, breathing strength, and the use of a face mask. Greater dispersion occurs when people sneeze, cough, and when they speak or breath harder while walking or running [12]. This greater dispersion distance implies that social distancing should be greater when people walk along the pavements. At this point, there is a gap in the literature regarding virus transmission control within the context of pedestrian mobility.

The goal of the present study was to evaluate the potential of pedestrian counters to monitor and control social distancing through a simple tool based on the classic measure of pedestrian level of service (PLOS). Pandemic pedestrian level of service (P-PLOS) has been designed to take more realistic measurements of pedestrian mobility in situations where specific recommendations on interpersonal or social distancing must be complied with. This tool could be of great use for decision making regarding tactical urbanism measures. This study, developed for the central district of the city of Madrid, had three main objectives, as follows:

1. Evaluate the effects of social distancing on spatio-temporal patterns of pedestrian mobility using pedestrian counters.
2. Propose a pedestrian level of service for pandemics that enables the use of tactical urban planning measures.
3. Evaluate the pedestrian level of service with and without social distancing.

The main contributions made by this study are: (1) rethinking and adapting PLOS to a situation with social distancing; (2) providing an example (in a contemporary context) of the use of pedestrian counter data; and (3) showing the results for the city centre of Madrid, where pedestrian counters have only recently been installed.

This article is structured as follows: Section 2 contains the literature review. Section 3 presents the data and methodology used to assess the spatio-temporal effects of the pandemic on pedestrian mobility. Next, in Section 4, we compare the spatio-temporal distribution of pedestrians in a year prior to the pandemic to the distribution in a year during the pandemic, and analyse the pandemic pedestrian level of service in the study area. Finally, in Section 5, we discuss the results obtained and the advantages of having pedestrian counters to allow for decisions to be made in real time based on pandemic pedestrian levels-of-service (P-PLOS).

## 2. Literature Review

### 2.1. Pedestrian Levels of Service (PLOS)

Levels-of-service (LOS) are useful tools for assessing the capacity of transport infrastructure [13] or a station to accommodate pedestrians or bicycles. In the first analyses of LOS, some authors used pedestrian density to evaluate capacity and space requirements in roadways [14]. Later, other researchers then applied different indicators to assess the level of service, such as the combination of the pavement section and pedestrian density [15]. It is clear that there has been a methodological enrichment in the calculation of LOS. The great advantage of LOS as a tool is that the representation of the results takes place on a scale (from A to F) that is easy to understand. As a result, it has become common in planning and decision making, as reflected in its frequent appearance in reference manuals [16].

However, in a situation of normality without restrictions on mobility, pavements are infrastructures on which people are in movement. They may be characterised by the needs of the pedestrians with respect to the structure, safety, comfort, and attractiveness of the street [17]. These characteristics of the street create incentives or disincentives for pedestrian movement on them and encourage or limit interactions with existing shops or other pedestrians. This implies that the choice of itinerary by pedestrians from a point of origin to a specific destination may be different according to the pedestrian quality of the environment [18]. Taking into account these circumstances, it is essential to know how pedestrians are distributed in space and time for the purpose of making decisions, particularly when there is a high pedestrian flow rate, which may have a negative impact on social distancing. Data from pedestrian counters, among other sources (see Section 2.2.), can play a key role. In addition, in recent years, numerous attempts have been made to simulate pedestrian mobility [19,20], and specific simulation tools have been developed on the basis of the PLOS in the context of social distancing [21]. These simulation tools could be enriched or validated by real data from pedestrian counters.

### 2.2. Big Data

In recent years, there has been a revolution in the use of geolocation data [22] for different forms of transport, providing us with a better understanding of movements in cities [23]. In addition, these data from geolocated devices and sensors provide a valuable source of information for pandemic monitoring and control [24], allowing for the identification of spatial transmission [25].

The information generated is very heterogeneous, depending on the type of geolocated device. In the case of fixed devices, the information generated does not allow for the reconstruction of trajectories or to have a user profile. However, when the device is individual (such as a mobile phone, shared bicycle, transport card, or credit card), the information generated allows for the reconstruction of spatio-temporal trajectories of the person. This type of device also allows for better user characterisation, as it is usually accompanied by socio-demographic data (age, gender, etc.). This means that mobile phone data, for example, can assist in the modelling of the geographical spread of epidemics [26,27].

Beyond mobile phone data, the construction of a big data information system for epidemics has been limited by multiple challenges, such as data acquisition, data integration, or multi-scale dynamics, among others [25]. These challenges are particularly notable with regard to pedestrian mobility. The data acquisition for pedestrian mobility continues to be limited and restricted, both by technology and location. In response to this situation, collaborative initiatives, such as WeCount (https://we-count.net/ (accessed on 5 April 2021)), are beginning to emerge and are of great interest. This collaborative project can carry out mobility and air quality measures in a host of streets throughout Europe.

Despite the limited data, there are some examples that assist in the acquisition of big data, such as the data collected by Apple [28], although its level of data disaggregation does not allow for the analysis of changes at a detailed level (neighbourhood, census district, or street). Using mobile phones as a source, Hunter et al. [29] analysed the change in the behaviour of the population of the United States with respect to walking, assessing the variations in time and distance before and after the pandemic. However, the use of this source of data has its limitations. First, the source used provided a great volume of data, which have to be filtered to obtain data that correspond to pedestrian mobility according to the criteria. Second, the data from mobile phones obviously do not include information on people who walk without their mobile phone [29] or who carry it without being connected. One method of overcoming these problems is to use data generated by fixed sensors, such as pedestrian counters. These counters provide data on the number of people who pass by a particular point. The disadvantage, in this case, is twofold. Firstly, a large number of such counters have to be deployed to obtain data from the whole city, something that rarely happens. Secondly, the counters do not measure the socio-demographic attributes of the people passing. Nevertheless, the lack of socio-demographic information is not a determinant in our application, since P-PLOS focuses on the flow of people passing through a given area to decide whether, at a given moment, this flow may entail risk.

### 2.3. COVID-19 Transmission

Social distancing is a response to the significant capacity of the virus to be dispersed through the air. There are many articles and pre-prints providing information on the dispersal of COVID-19, which is closely related to particle size and the force of exhalation [30,31] when talking, coughing, or sneezing. Based on all the scientific information generated, including not only that related to COVID-19 but for other infectious diseases transmitted by air as well, public health organisations at both global and national levels formulated the recommendation to maintain a social distance of between 1 and 2 m [32]. The World Health Organisation (WHO) [33] recommends a distance of at least 1 m. The Centre for Disease Control and Prevention (CDC) [34] proposes 6 feet (about 1.8 m), and the Spanish Ministry for Health recommends a distance of 2 m and the use of masks when a separation of 1.5 m cannot be maintained [35].

Some studies were also carried out, such as the one by Córdoba-Hernández et al. [36], who calculated the relationship between the area of the pavement and the population, demonstrating the zones that are deficient with respect to the area of pavements. However, although this information could be of interest, it merely demonstrates the deficit in pedestrian infrastructures with respect to the population and social distancing, in which people are considered non-mobile elements who occupy space on pavements in a static situation. A static scenario like this may be of some use to highlight the need for more pedestrian space to prevent virus transmission, but only in situations where movements are restricted by space and time [36], as implemented in Spain from 1 May 2020 [37].

Therefore, it is essential to consider a dynamic scenario of pedestrian mobility, as it is only in these kinds of scenarios that variables that influence the dispersion of the virus and that derive from a subject in motion with more intense exhalations can be included. For the scenario of pedestrian mobility, Blocken et al. [38] proposed a distance of 5 m when walking, 10 m when running, and 20 m in the case of bicycles.

## 3. Materials and Methods

### 3.1. Case Study

**Figure 1:**
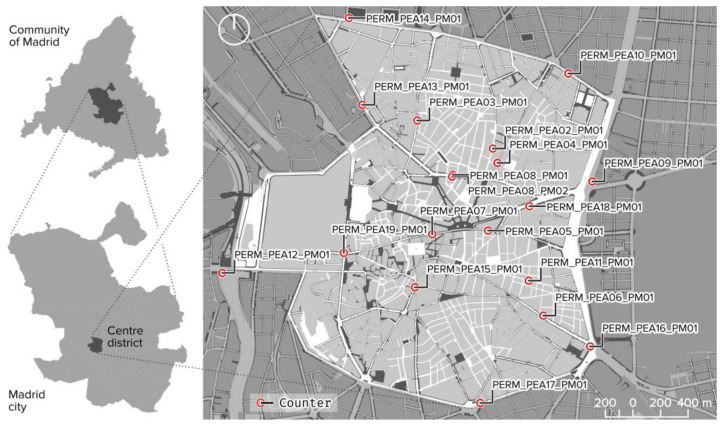
Study area location. Source: Data from [27] and OpenStreetMap contributors.

To assess the effect of the pandemic on pedestrian mobility, we used the city of Madrid as a case study, more specifically the Centro district (Figure 1). In particular, the Centro district is characterised by its strong cultural component, which makes it a district with a large presence of tourists from all countries, whose digital footprints have previously been measured by García-Palomares et al. [39] and Salas-Olmedo et al. [40]. This distinctive feature results in a predominance of commercial premises (36.8%) and hotels and restaurants (25.5%) according to Madrid’s retail census obtained from the open data website [41].

From the point of view of mobility, in 2018, the Centro district joined the low-emission zone, Madrid Central, which promotes sustainable transport, such as walking and cycling, and where public transport is favoured over private transport.

In short, it is a district with a high flow of pedestrians compared to other areas, which, in turn, makes it an area where crowding is more likely to cause risky situations. As Madrid is a big city, the amount of data generated at the private and public levels is greater than in other cities, which makes it easier to test the proposed tool. The City Council of Madrid generates a multitude of data relating to mobility (traffic, bicycles, pedestrians, etc.), which it makes available to the public through its open data website (https://datos.madrid.es/portal/site/egob (accessed on 5 April 2021)).

### 3.2. Data

The main source of data used to assess the effect of the pandemic on pedestrian mobility has been the pedestrian counters installed in the city of Madrid. To compare trends, we used other mobility data during COVID-19 provided by Apple and Google.

The characteristics of these sources of data are specified below:

(a) The data provided by Apple [28] refer to the number of requests for directions by country, region, or city, and were compared to the reference data of 13 January 2020 used in this study. These data from Apple users were included anonymously, which implies that the associated identifiers are random and rotating. Moreover, the data do not include any demographic information about the users, so no relations can be established with specific population groups.
(b) Google provided Local Mobility Reports on COVID-19 [42], which showed the trend in movement over time at different scales, classified by categories of places (shops and leisure, supermarkets and pharmacies, public transport stations, workplaces, and residential areas).
(c) The database of pedestrian counters is available to the public on the Madrid Council’s open data (Datos Abiertos) website [41]. This data source is maintained by the local administration, which periodically publishes data with a high level of disaggregation and in an exhaustive manner, which results in high-quality data. The counter data have been available since 2019, but they are not homogenous, and these limitations must be taken into account if the information is to be correctly processed. A total of 19 counters are available and distributed around the Centro district, some in main streets and others in smaller, more outlying ones. The durations for which the numbers of pedestrians were recorded was 15 min in 2019 and 60 min in 2020 and 2021. This change in frequency must be taken into account and we return to it in Section 3.3.
(d) Finally, we used the street plan of the pavements of the city of Madrid as the base map for analysis. It is freely available through the Geoportal of the Madrid Council [43] at a scale of 1:1000, with the latest update in 2016. This information has been updated manually on the pavements that have been extended after the date of publication of the plan.

### 3.3. Methodology

**Figure 2:**
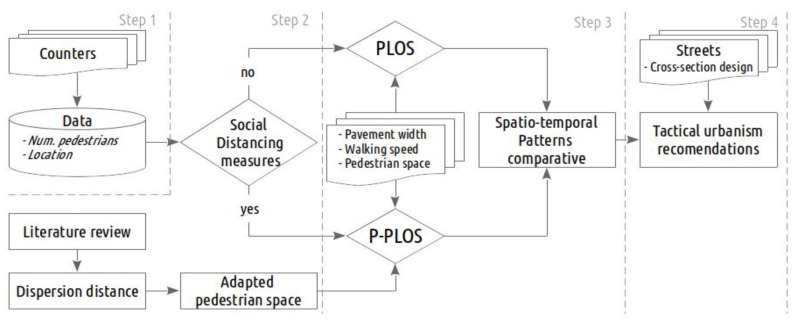
Methodology diagram.

To apply a new pedestrian level of service for a pandemic that can be used together with tactical urbanism to improve pedestrian flows and reduce health risks, we propose a specific methodology (Figure 2) that has been structured into 4 main steps.

The first step is fairly technical and includes data preprocessing and street characterisation.

The second step entails an overall vision of the effects of the restrictive measures related to COVID-19 on pedestrian mobility.

The third step proposes a pedestrian level of service, which allows for an assessment to be made of the spatio-temporal patterns in which pedestrian flows are high and, therefore, involve a risk of contagion.

In the final step, the pedestrian level of service is analysed as a whole, together with the layout of the street, so that recommendations for action can be prepared.

#### 3.3.1. Data Preprocessing and Street Characterisation

We have written a script in the Python programming language that allows for the downloading of information in text format (csv) from the websites of Google, Apple, and Open Data of Madrid, checking for errors and missing data.

From January to June 2019, the pedestrian counters in Madrid were at a testing phase, so the data included may be overestimated or underestimated. In addition, the location of the counters in this first six-month period was not final, making it difficult, in some cases, to compare the later data. Finally, the frequency of counting also varied. Although the Council’s website suggests that the counting was carried out every 15 min, this frequency was only used in the testing phase; after that, the frequency was every 60 min. Moreover, data were unavailable in July and August 2019 due to a malfunction in the sensors. For all the above reasons, we eliminated all the data before September 2019. This article only covers the period from September 2019 to April 2021.

The text files in csv format from Apple and Google were filtered by the Python Pandas library to obtain the information for Madrid, and the format was homogenised to allow for comparison.

The area of the pavements was calculated according to the information provided by the Madrid Council, with the data being updated for the pavements whose width was extended after the publication date. After updating the geometry of the streets, their width at the point where the counter was located was obtained.

Table 1 shows a brief description of the counter number, the street, the street number at which the sensor is located, and the pavement width. Further details on the characteristics of the section with respect to the modal distribution are included in Appendix A. The counters capture the information on the pavement where they are located, which are distinguished with respect to the number of doorways to homes and the side of the street (odd or even street numbers). In the case of pedestrianised streets, the sensor refers to the whole section.

#### 3.3.2. Spatio-Temporal Patterns

Two periods were used to analyse the changes in the temporal patterns of pedestrian mobility.

First, the temporal patterns were analysed at a general level, comparing the data from Google, Apple, and the data on pedestrian counters. For this initial analysis, the data offered by Apple and Google were used, covering the period between January 2020 and April 2021. This analysis only allows for daily change details due to the level of detail of the Google and Apple data.

The level of detail offered by the data from the counters was examined and a comparative analysis was carried out on pedestrian mobility in the third four-month period (September to December) of the years 2019 and 2020. These periods of time provide a broader vision with which to compare a normal period and a period of “new normality”, in which there were special restrictions on mobility in specific zones and times. In addition, we analysed the patterns of pedestrian mobility in terms of space by a disaggregated use of the counters. This disaggregated use of the data by the counters allowed us to determine the impact of mobility related to the characteristics of the streets in which the counters are located

#### 3.3.3. Designing a Pandemic Pedestrian Level of Service (P-PLOS)

Once the spatio-temporal patterns of pedestrian mobility during the pandemic were analysed, the pedestrian level of service tool was redesigned to adapt it to a pandemic context, taking into account the recommendations of the health authorities and scientific studies published to date.

**Figure 3:**
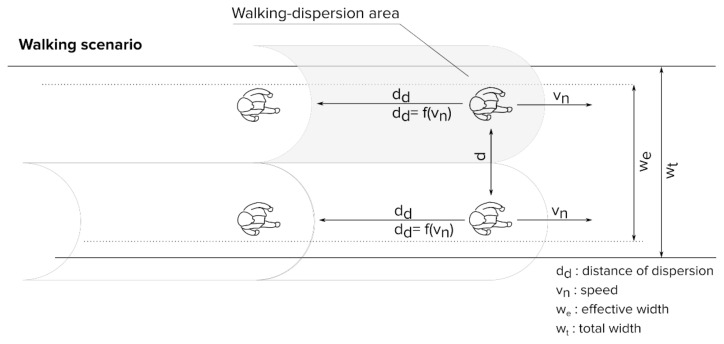
Proposal of social distancing for the walking scenario.

To calculate the pandemic pedestrian level of service, the variables of the walking scenario had to be established based on the level of social distancing when the population is stationary (Figure 3), which were set at a lateral distance of 1.5 m, according to the recommended interpersonal distance. In movement, the dispersion is mainly produced along the displacement axis. The work by Blocken et al. [38] on the safety distance for aerosol transmission as related to the speed of walking was used as a basis for this work. We established the same walking speed of 4 km/h, and a safety distance of a minimum of 5 m, as a reference. The distance used was the distance at which the minimum presence of particles is detected as expelled when breathing by a person walking at a speed of 4 km/h [38].

Using the above data, we calculated the walking-dispersion area, which was used as a reference for the level of service:

(1) $$A_{d}=D_{d}f\left(v\right)*LD_{d}$$

where:

*A_d_* is the walking-dispersion area;
*D_d_f(v)* is the walking-dispersion distance based on speed;
*LD_d_* is the walking-dispersion lateral distance.

Following this formula, the walking dispersion area at 4 km/h is 7.5 m^2^ (5 m distance * 1.5 m of lateral distance).

Then, the values corresponding to the pedestrian service levels were recalculated (Table 2) based on the original pedestrian service level tool in the Highway Capacity Manual 2000 [16] and the following formula:

(2) $$V_{p}=\frac{S_{pr}}{A_{p}}$$

where:

*V_p_* is the flow rate per unit of width (pedestrian/min/m);
*S_pr_* is the reference pedestrian walking speed (m/min);
*A_p_* is the pedestrian space (m^2^/p).

The width of the pavement for the purpose of measuring pedestrian flow takes into account only the effective width of the actual pedestrian pavement. There are many features along the pavement that may be an obstacle to people and that reduce the pavement width. These obstacles can be fixed, such as signals, streetlamps, some types of tree surrounds, benches, and other types of urban furniture. Other features include temporary obstacles, such as restaurant or shop banners near facades, people exiting from building entries, window shoppers, parked scooters, and so forth. Whereas fixed obstacles are easy to measure, measuring the pavement width considering temporary obstacles may be more difficult and may involve a high cost in terms of personnel and time.

Given all of the above, and following the research undertaken by Córdoba-Hernández et al. [36], we consider that the effective width represents 55% of the total pavement.

## 4. Results

### 4.1. Changes in Mobility Patterns

**Figure 4:**
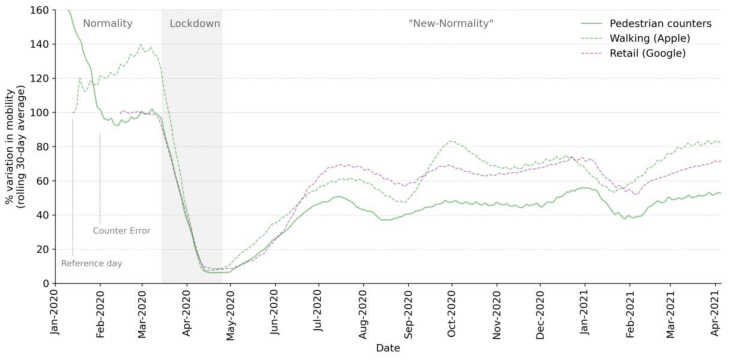
Variation (%) in the number of pedestrians from the reference day.

The effect of the COVID-19 pandemic on pedestrian mobility in Madrid (Figure 4) shows how the confinement measures of the first wave of the pandemic (March 2020) led to a swift fall (94% from the reference day) in the number of pedestrians recorded by the counters. This limited number of pedestrians was maintained until May, when the measures were eased, allowing people to walk in the open air. As the conditions were progressively eased, the number of pedestrians recorded increased until the summer months (July and August), when people left Madrid on holiday and there were no arrivals of foreign visitors. With the return of people from their holidays and the start of the school year, there was a slight increase in the number of pedestrians, which was maintained, except for a few weeks, until the end of 2020. The data recorded for the first quarter of 2021 were stable with respect to the number of pedestrians recorded

When comparing the data recorded by the counters to the data provided by Apple for the analysis of mobility during the pandemic, it can be seen how the temporal pattern is similar, in general (Figure 4). However, there are certain differences in some periods of time due to the scales of the samplings. In the case of Apple, the data cover the whole city of Madrid, while the counters are located solely in the Centro district, so they may record a lower level of pedestrian mobility.

**Figure 5:**
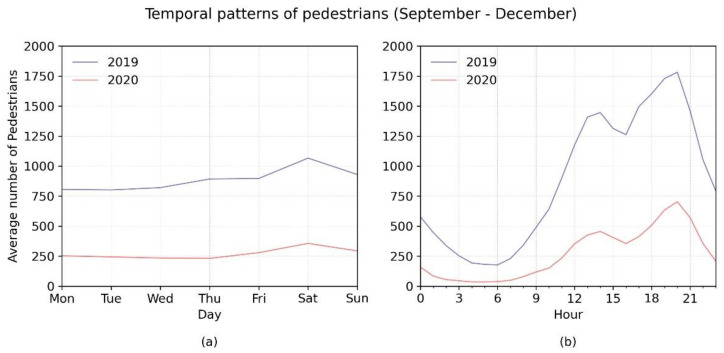
Temporal patterns. (**a**) Day of week, (**b**) time of day.

The comparison between the third quarters of 2019 and 2020 shows very similar patterns with respect to the distribution of pedestrians on the days of the week (Figure 5a), where there were peaks in the numbers of pedestrians on Saturdays. With respect to the distribution of pedestrians at different times of the day (Figure 5b), and taking into account the significant reduction in the number of pedestrians, the temporal distribution remained the same, with a peak between 13.00 and 14.00 h, and a second peak with a larger number of pedestrians at 20.00 h.

**Figure 6:**
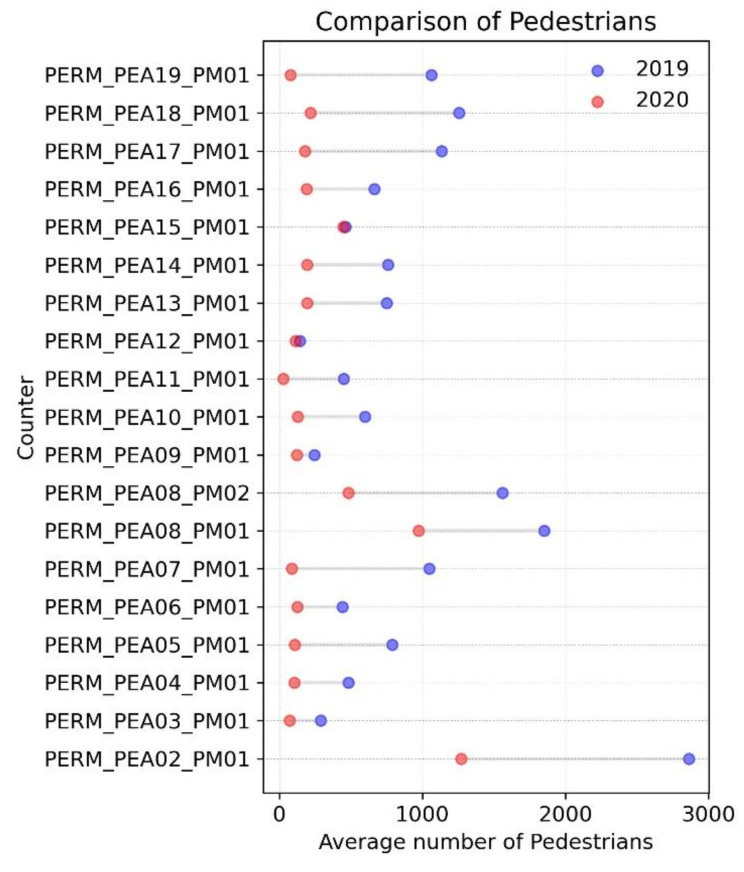
Change in the number of pedestrians by counter station.

Once the results showing the overall number of pedestrians were determined, it was interesting to investigate the temporal patterns by counter to detect differences in the spatio-temporal patterns. Figure 6 shows how the measures and recommendations on mobility have had a different impact depending on the characteristics of the street. The reduction in the number of pedestrians recorded by each of the counters fell considerably, with similar values being recorded in the periods under analysis (except for the counter PEA15). It is worth noting that the counter PEA02, despite showing the biggest fall in the average number of pedestrians, registered values in 2020 at above 1000 pedestrians/hour.

**Figure 7:**
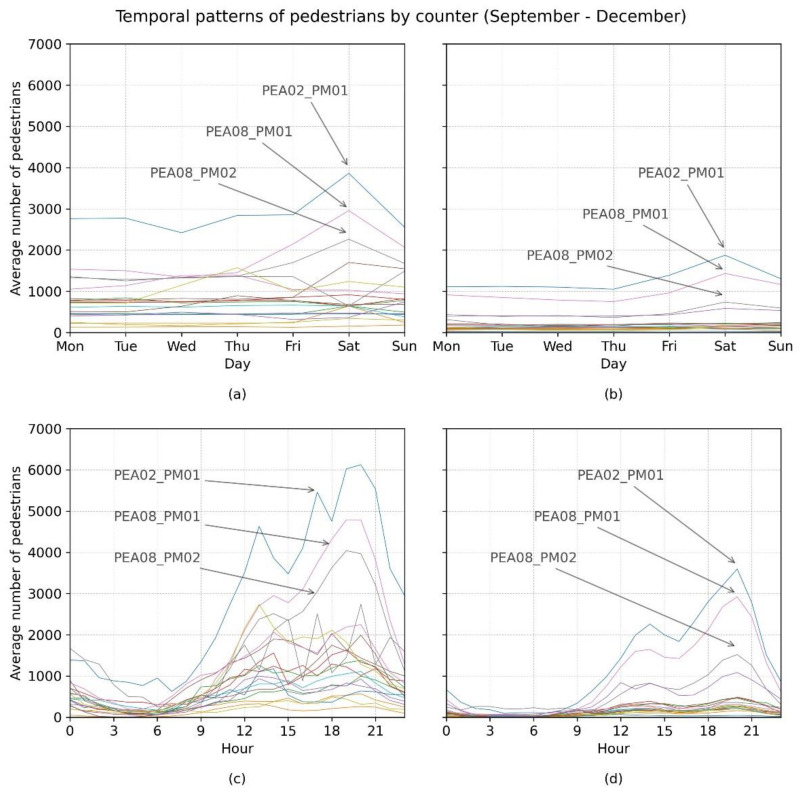
Temporal patterns by counter (**a**) day of week 2019, (**b**) day of week 2020, (**c**) time of day 2019, (**d**) time of day 2020.

Looking in more detail at the temporal distribution by pedestrian counter (Figure 7), clear differences can be seen in the numbers of pedestrians passing by the different counters. These differences are clear for 2019 and even more so for 2020. In both years, three particular counters registered the greatest number of pedestrians: PEA02-PM01, followed by PEA08-PM01 and PEA08-PM02.

### 4.2. Changes in Pedestrian Level of Service (PLOS)

**Figure 8:**
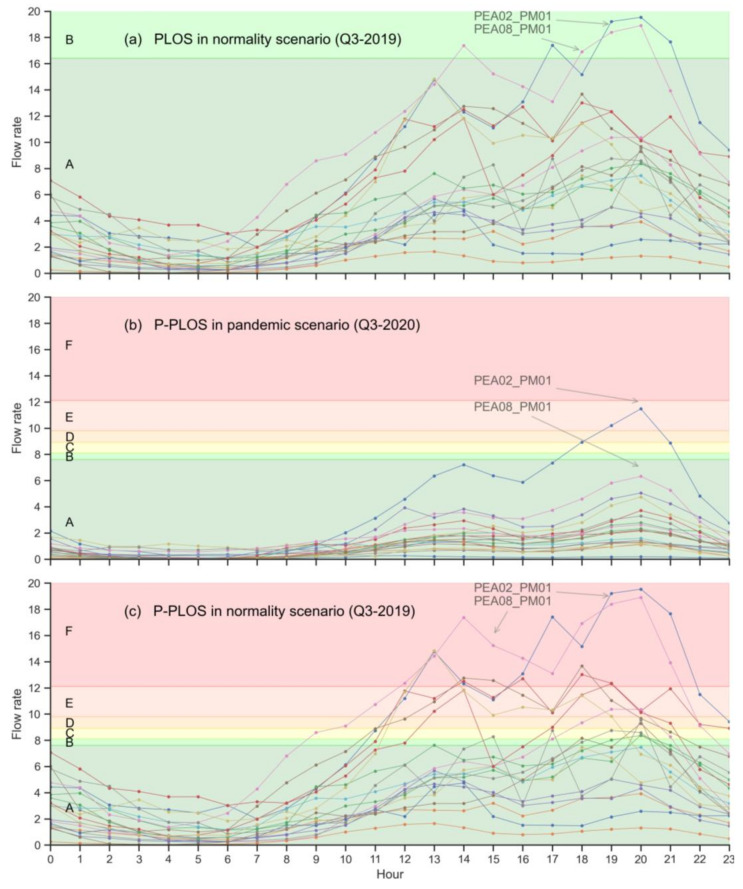
Comparison of the pedestrian levels of service in normality scenario (**a**), pandemic scenario (**b**) and P-PLOS in normality scenario (**c**).

The results of applying service levels with the data collected (Figure 8) show how for a scenario of normality, the average values of the pedestrian level of service (PLOS) for the period under analysis were at optimal levels (Level A) only for counters PEA02-PM01 and PEA08-PM01 at certain times of the day. For its part, PEA08-PM01 registered a fall in the level of service between 17:00 h and 21:00 h.

Regarding the pedestrian levels-of-service during the pandemic (P-PLOS) (Figure 8b), it is clear that the recommended social distancing measures have had an obvious effect on the levels of service. In general, there is a great homogeneity in the levels of service in 2020, for which the peaks of mobility were at the usual times (from 13:00 to 15:00 h and from 18:00 to 21:00 h). This homogeneity was not reflected in the counter PEA02-PM01, which registered the larger number of pedestrians and gave rise to low levels-of-service (D, E) where interpersonal and social distances were recommended, or the speeds were reduced.

Finally, it is worth analysing the results of the normal scenario from the pandemic level of service perspective (Figure 8c). This scenario shows how the counters PEA02-PM01 and PEA08-PM01, located in the streets Fuencarral and Gran Vía, respectively, were at level of service F, which is associated with pedestrian flows in which it is difficult to maintain a safe distance.

## 5. Discussion

COVID-19 has had a major impact on mobility, both through a change in mobility patterns [6] and changes in public attitudes [44]. During this time, several adaptation actions have emerged, such as the implementation of pop-up bike lanes, among other measures [7]. In addition, this pandemic has put transport systems to the test, showing their weaknesses and strengths, and allowing new challenges to be faced for more sustainable mobility [45].

Pedestrian mobility has probably been impacted the most, and it is clear that the pedestrian levels of service that have been so widely used in pedestrian mobility analysis cannot be directly applied in situations with social distancing. At this point, there are two main challenges that need to be met: the need to consider a dynamic scenario (pedestrians on the move) that takes into account the spread of the virus; and the need for pedestrian count data, preferably real and in real or quasi-real time. This would allow decisions to be made once decreases in the pedestrian levels of service are detected.

In this context, we have proposed the pandemic level of service (P-PLOS), whereby an adjustment of levels of service is made considering situations with social distancing, and which is fed by pedestrian flow data taken from pedestrian counters. P-PLOS has been tested for the case of the central district of Madrid. The city of Madrid has recently started publishing data from these pedestrian counters (September 2019), but no previous works have used this data yet. The results obtained show that the measures restricting mobility have been useful for avoiding agglomerations (and would have, at least theoretically, produced a lower propagation of the virus outdoors). Once the restrictive measures were eased, pedestrian mobility increased. However, this recovery in pedestrian mobility was not the same in all the streets with counters. In streets with many commercial uses, the recovery was greater due to their attractiveness for pedestrians [46], while in streets without commercial uses, the presence of pedestrians has remained low.

At the same time, the measures taken in Madrid to restrict mobility at night had an effect on the presence of pedestrians in the early hours, but it did not represent a modification in the temporal pattern (Figure 7).

However, beyond the minor presence of pedestrians in the streets under analysis, it is necessary to analyse this presence in terms of the pedestrian level of service, taking into account the recommended safe distance.

From the perspective of the pedestrian level of service during the pandemic, it can be seen that the streets with significant retail activity, such as Fuencarral and Gran Vía, have low (E) or very low (F) levels of service, mostly in the afternoon. These low levels of service also occurred on the dates closest to Christmas, which is traditionally known as the Christmas shopping period. According to the results obtained, at these peak hours, with high numbers of pedestrians, distances and speed were affected, increasing the risk of not maintaining the safe distance.

It is in these situations in which the level of service declines that tactical urbanism can play a decisive role. Tactical urbanism allows for action to be taken according to the characteristics of the street to allow for a greater proportion of the pavement to be used by pedestrians [47,48]. Thus, in cases in which the street has a parking lane, this lane may be adapted for pedestrian use to ensure the maintenance of a good pedestrian level of service. In streets where there is no parking lane, but there are a number of lanes of traffic in the same direction (as in the case of Gran Vía), one of them could be used for pedestrians to move in the hours with the greatest expected presence. Finally, in cases in which the street is pedestrianised (such as Fuencarral), actions should be taken to limit the presence of pedestrians to reduce the risk of contagion. These deterrent measures may take various forms, ranging from restricting access to the street or redirecting these pedestrians to other streets. Of course, some of these actions have been put in place by local governments at certain times during the pandemic, but P-PLOS can help them to make better more informed decisions considering local situations and needs.

This research also makes clear the usefulness of introducing sensors in cities, as they allow for monitoring, in real or quasi-real time, of a number of aspects of the city, from air quality to mobility, as in the case we investigated. These sensors, which generate a large quantity of data, may be very useful in decision making and the management of mobility in a variety of circumstances. This tool allows for active management of pedestrian mobility, maintaining the distances recommended by the health authorities, and providing appropriate information for tactical measures in certain streets and at specific times during the week or in the day.

Among the main limitations of this study are the levels of disaggregation of the data from the different sources. In the case of Apple and Google, there was a limitation of scale that prevented us from knowing the changes in mobility by district or census zone, as well as the fact that the interval between data collections was a day. With respect to the pedestrian counters, there is a limitation in the sample interval, which was increased from 15 min (recommended level) to intervals of 1 h. It should also be noted that the sensors are located in the Centro district. Information on other districts in the city is, therefore, not available for appropriate action to be taken. However, it would be possible to introduce data from other sensors, such as temporary or specific pedestrian time sensors, including the WeCount initiative. In any case, it is precisely the central district where the highest pedestrian flows in the city of Madrid occur [49], making it a strategic area for monitoring possible risk situations. The absence of socio-demographic information on pedestrians could also be seen as a limitation. Of course, this information would be useful for a better characterisation of the flow of pedestrians, e.g., to adjust walking speeds and even to assess the intrinsic vulnerability of pedestrians. However, we believe that P-PLOS can enable the effective monitoring of the flow of pedestrians and be the basis for further analysis and applications.

Finally, it is worth clarifying that this study used the distance recommended by the Spanish authorities as the safe distance. However, the measurement of the pedestrian level of service in times of the pandemic can be easily modified to adapt it to the requirements of the health authorities in other countries and regions. Moreover, the measures recommended with respect to the use of masks should be taken into account, as the risk of contagion could vary significantly. Clearly, P-PLOS has proved to be useful considering the restrictions arising from the COVID-19 pandemic, but it can be applied in any situation where a certain safe distance has to be maintained.

## 6. Conclusions

The social distancing measures, in their different levels of severity, have had a notable impact on mobility, although their effects on pedestrian mobility have, so far, not been extensively analysed.

This article proposes an adaptation of the measurement of pedestrian level of service (PLOS), which we named the pandemic pedestrian level of service (P-PLOS), to incorporate the recommendations on interpersonal distancing levels. The data for the calculation are obtained from pedestrian counters and allow us to assess the service level of a segment of a street and, thus, assess in real or quasi-real time whether there are situations of potential risk when the interpersonal distance is reduced. The results of P-PLOS can assist with the development of health and active mobility policies by providing information on the need for tactical urbanism interventions in the locations and at the times when the levels of service are most deficient. This alliance between sensors and tactical urbanism would allow planners to modify the modal distribution of the streets to achieve more sustainable and safer mobility.

## Figures and Tables

**Table 1:** Description of pedestrian-counter locations.

Counter Name	Street	Building No.	Description	Pavement Width (m)
PERM_PEA02_PM01	Calle Fuencarral	22	Pedestrian street	9.5
PERM_PEA03_PM01	Calle San Bernardo	36	Even numbers	3
PERM_PEA04_PM01	Calle Hortaleza	18	Even numbers	3.6
PERM_PEA05_PM01	Carrera de San Jerónimo	6	Even numbers	3
PERM_PEA06_PM01	Calle Atocha	95	Odd numbers	5
PERM_PEA07_PM01	Calle Mayor	13	Odd numbers	4.5
PERM_PEA08_PM01	Gran Vía	34	Even numbers	14
PERM_PEA08_PM02	Gran Vía	33	Odd numbers	14
PERM_PEA09_PM01	Paseo de Recoletos	22	Even numbers	2
PERM_PEA10_PM01	Calle Génova	12	Even numbers	4.5
PERM_PEA11_PM01	Calle Huertas	29	Pedestrian street	7.5
PERM_PEA12_PM01	Madrid Río ^1^	1	Pedestrian street	7.5
PERM_PEA13_PM01	Calle Princesa	1	Odd numbers	5
PERM_PEA14_PM01	Alberto Aguilera	56	Even numbers	3.6
PERM_PEA15_PM01	Calle Toledo	23	Odd numbers	6.3
PERM_PEA16_PM01	Plaza del Emperador Carlos V	11	Odd numbers	6.5
PERM_PEA17_PM01	Ronda de Valencia	16	Even numbers	3.6
PERM_PEA18_PM01	Calle Alcalá	34	Even numbers	4.5
PERM_PEA19_PM01	Calle Bailén	10	Even numbers	5.6

^1^ Puente de Segovia with Paseo Ermita del Santo.

**Table 2:** Comparative values of PLOS and P-PLOS.

Level of Service	PLOS (HCM, 2000)			P-PLOS		
	Space (m^2^/p)	Flow Rate (p/min/m)	Speed (m/min)	Space (m^2^/p)	Flow Rate (p/min/m)	Speed (m/min)
A	>5.6	<16.40	>77.72	>8.8	<7.6	>83.3
B	5.6–3.7	16.40–22.97	77.72– 76.26	8.8–8.2	7.6–8.1	83.3–75.0
C	3.7–2.2	22.97–32.81	76.26– 73.15	8.2–7.5	8.1–8.9	75.0–66.7
D	2.2–1.4	32.81– 49.21	73.15–68.58	7.5–6.8	8.9–9.8	66.7–58.3
E	1.4–0.75	49.21–75.46	68.58– 45.72	6.8–5.5	9.8–12.1	58.3–41.67
F	<0.75	>75.46	<45.72	<5.5	>12.1	<41.67

## Data Availability

Further data is available at: https://datos.madrid.es/egob/catalogo/300321-1-aforos-peatones-bicicletas.csv (accessed on 5 April 2021); https://datos.madrid.es/egob/catalogo/300321-4-aforos-peatones-bicicletas.csv (accessed on 5 April 2021); https://datos.madrid.es/egob/catalogo/300321-8-aforos-peatones-bicicletas.csv (accessed on 5 April 2021).

## Appendix A

**Table A1:** Street characterisation of pedestrian counter locations.

Counter name	Pavement Width (m)	Parking lane	>1 Traffic Lane	Pedestrian Flow (Peak Hour) ^1^	Photo
PERM_PEA02_PM01	9.5	No	No	6124 (19–20 h)	
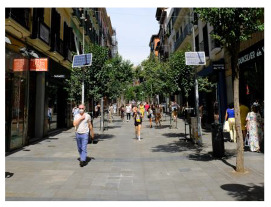
PERM_PEA03_PM01	3	Yes	No	570 (19–20 h)	
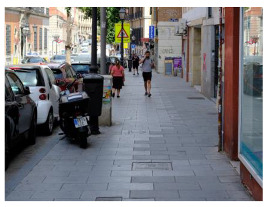
PERM_PEA04_PM01	3.6	No	No	997 (19–20 h)	
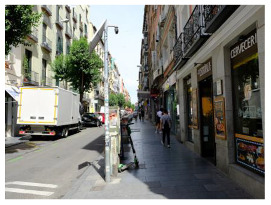
PERM_PEA05_PM01	3	No	No	1288 (17–18 h)	
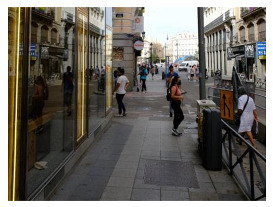
PERM_PEA06_PM01	5	No	Yes	937 (12–13 h)	
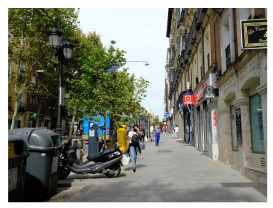
PERM_PEA07_PM01	4.5	Yes	No	2031 (17–18 h)	
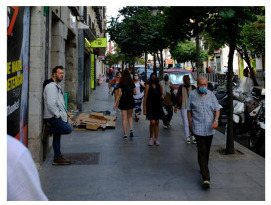
PERM_PEA08_PM01	14	No	Yes	4787 (18–19 h)	
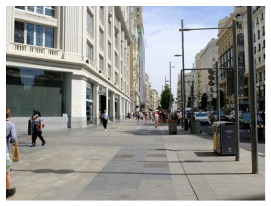
PERM_PEA08_PM02	14	No	Yes	4041 (18–19 h)	
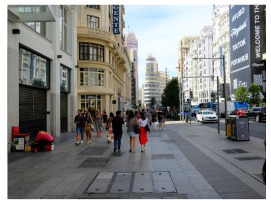
PERM_PEA09_PM01	2	No	Yes	493 (17–18 h)	
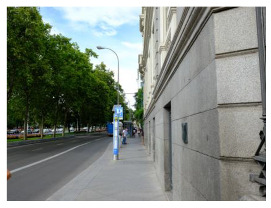
PERM_PEA10_PM01	4.5	Yes	Yes	1108 (19–20 h)	
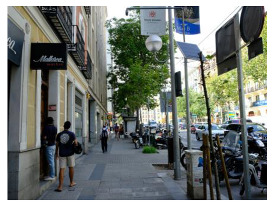
PERM_PEA11_PM01	7.5	No	No	1172 (13–14 h)	
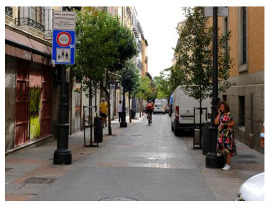
PERM_PEA12_PM01	6	No	Yes	328 (12–13 h)	
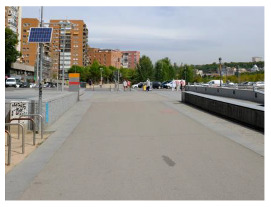
PERM_PEA13_PM01	5	No	Yes	1380 (19–20 h)	
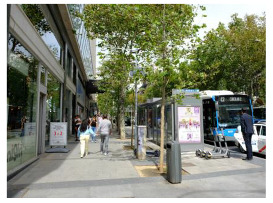
PERM_PEA14_PM01	4	No	Yes	1626 (18–19 h)	
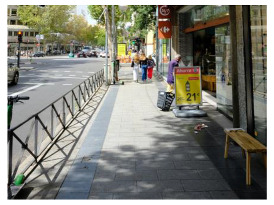
PERM_PEA15_PM01	6.3	No	No	999 (12–13 h)	
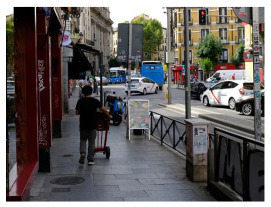
PERM_PEA16_PM01	6.5	No	Yes	1992 (19–20 h)	
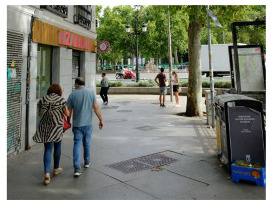
PERM_PEA17_PM01	3.6	No	Yes	2246 (19–20 h)	
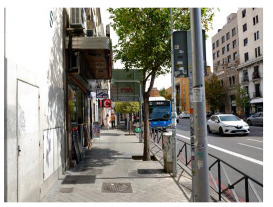
PERM_PEA18_PM01	4.5	No	Yes	2740 (19–20 h)	
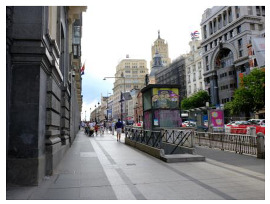
PERM_PEA19_PM01	5.6	No	Yes	2740 (12–13 h)	
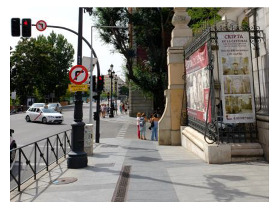

^1^ Peak of pedestrian flow (ped/hour) before COVID-19 (Sep-Dec 2019) and peak hour interval.
